# Enhancing Chitosan Fibers: A Dual Approach with Tripolyphosphate and Ursolic Acid

**DOI:** 10.3390/polym16040461

**Published:** 2024-02-07

**Authors:** César I. Hérnandez Vázquez, Zbigniew Draczyński, Dominik Borkowski, Dorota Kaźmierczak

**Affiliations:** 1 Institute of Materials Science of Textiles and Polymer Composites, Lodz University of Technology, 116 Żeromskiego, 90-924 Lodz, Poland; zbigniew.draczynski@p.lodz.pl (Z.D.);; 2 Łukasiewicz Research Network-Lodz Institute of Technology, 19/27 M. Skłodowskiej-Curie Str., 90-570 Lodz, Poland

**Keywords:** chitosan, fibers, ursolic acid, antibacterial, chitosan fibers, tripolyphosphate

## Abstract

Chitosan, a well-established biomaterial known for its biocompatibility, biodegradability, and bioactivity, has been the focus of extensive research in recent years. This study explores the enhancement of chitosan fibers’ properties through wet impregnation with either ursolic acid (UA) or cross-linking with tripolyphosphate (TPP). In the first experiment, chitosan fibers were treated with UA, for varying immersion set points (1, 2, 4, 6, and 8 h). FTIR, SEM, and UV-Vis spectroscopy analyses demonstrated a chemical reaction between chitosan and UA, with stability reached after 2 h of immersion. Antibacterial testing revealed that chitosan fibers impregnated with UA exhibited significant antibacterial activity against Gram-positive bacteria, notably *Staphylococcus aureus*. The second experiment involved modifying chitosan fibers’ surfaces with a 1% *w*/*v* TPP solution for the same periods of time (1, 2, 4, 6, and 8 h). Subsequently, the investigation involved FTIR, SEM, and dynamometry analyses, which revealed successful cross-linking between chitosan and TPP ions, resulting in improved tensile strength after 2 h of immersion. This dual-approach study highlights the potential of chitosan fibers for diverse applications, from wound-healing dressings to antibacterial materials against Gram-positive bacteria.

## 1. Introduction

Among biomaterials, chitosan has emerged as a promising candidate for various biomedical applications, due to its multiple properties. The use of chitosan in textiles has been widely investigated by many researchers, with antimicrobial, water absorption, and tensile properties being some of the most important ones to study [1,2,3,4,5]. Chitosan is a biopolymer that possesses a range of valuable physicochemical properties such as solubility, reactivity, adsorption, and crystallinity. Additionally, it exhibits various biological properties including biodegradability, antimicrobial activity, cytocompatibility, nontoxicity, and fungicidal effects. Moreover, its beneficial characteristics, like anti-cholesteric and antioxidant activity, macrophage activation, anti-inflammatory effects, the stimulation of angiogenesis, mucoadhesion, antitumor properties, the promotion of granulation and scar formation, hemostatic action, and the facilitation of wound healing, position it as an exceptionally promising material for a multitude of applications in the field of biomedicine [6,7,8]. Chitosan is a linear polysaccharide that is composed of randomly distributed β-(1→4)-linked D-glucosamine and N-acetyl-D-glucosamine units [9]. The complexity of chitosan chemistry is influenced by various factors, which comprise the degree of deacetylation, molecular weight, and solution pH. The degree of deacetylation is the proportion of N-acetyl-D-glucosamine units that have been transformed into D-glucosamine units. This factor significantly impacts the solubility, reactivity, and charge density of chitosan [10]. In recent studies, chitosan fibers have been employed as a method of reinforcement to enhance the mechanical properties of scaffolds. The mechanical properties of the fibers play a vital role in determining the overall mechanical characteristics of the scaffold. Notably, chitosan scaffolds reinforced with high-mechanical-property chitosan fibers exhibited a doubling in strength and a fivefold increase in stiffness compared to scaffolds reinforced with fibers possessing lower mechanical properties [11,12,13,14].

Cross-linking is one of the processes applicable to chitosan for the purpose of enhancing its mechanical characteristics. Chemical cross-linking refers to the intermolecular or intramolecular joining of two or more molecules by a covalent bond. The reagents that are used for the purpose are referred to as ‘cross-linking reagents’ or ‘cross-linkers’. Physical and chemical cross-linking agents are the two main categories of cross-linking agents. Glutaraldehyde, formaldehyde, tripolyphosphate, polyaspartic acid and sodium salts, are the most commonly used cross-linkers for chitosan. However, at certain concentrations, glutaraldehyde and formaldehyde are considered to be toxic and to raise health concerns and cause undesirable side effects. To overcome this problem, non-toxic cross-linkers such as genipin or TPP are used [15]. Chitosan’s distinctive properties make it suitable for a wide range of applications. Its ability to establish cross-linked networks is an essential aspect of its functionality. Chitosan can be cross-linked using a variety of agents. Chitosan cross-linkers which are commonly used include glutaraldehyde [16], tricarboxylic acid [17], genipin [18], tripolyphosphate (TPP) [19], and epoxy compounds [20]. Tripolyphosphate (TPP) and also sodium triphosphate (STP) or sodium tripolyphosphate (STPP) are inorganic compounds; they are the sodium salt of the polyphosphate penta-anion, which is the conjugate base of triphosphoric acid.

On the other hand, one of the most intriguing properties of chitosan is its antibacterial activity. However, there is still a need to enhance its antibacterial properties to further broaden its potential applications, and this is a commonly studied topic among researchers [21,22,23,24]. In the past, different compounds have been studied that can act synergistically with chitosan to create a more potent antibacterial agent, and also potentially offer a wider spectrum of antibacterial activity against different types of bacteria, such as silver nanoparticles [23,25], essential oils [26,27], quaternary salts [28] plant extracts [29], and ursolic acid [30], among others. Ursolic acid and chitosan adducts have been relatively less studied, especially chitosan fibers and ursolic acid compounds.

Ursolic acid is a pentacyclic triterpene acid that is commonly found in various traditional medicinal plants, fruits, and ornamental species. It is known to exhibit a broad range of biological activities, including anti-inflammatory, anticancer, hypoglycemic, antioxidant, and antibacterial properties. The compound has been extensively studied for its antibacterial effects, as have other pentacyclic triterpenes and their derivatives. In addition to its potential therapeutic applications, ursolic acid has also been found to prevent abdominal adiposity and exhibit cytotoxic activities, as well as antiprotozoal effects against Plasmodium falciparum [31,32,33].

The aim of this work was to investigate and to expand the knowledge on how to improve the properties of chitosan fibers, which can lead to the creation of better biomedical materials. Chitosan fibers have demonstrated potential in the area of tissue engineering, but their mechanical properties require enhancement to improve durability and functionality. Additionally, the incorporation of a broader range of agents to enhance antibacterial properties can be beneficial in preventing infections in medical implants and devices. The ultimate goal was to develop chitosan fibers with enhanced antibacterial and mechanical properties, with potential applications such as for tissue engineering or as wound healing materials. Hence, the research findings can have a significant impact on chitosan and its applications, particularly for wet-spun fibers.

In summary, this comprehensive assessment encompassed FTIR, UV-Vis spectroscopy, and SEM analyses to scrutinize the chemical and structural aspects of the modified chitosan fibers. It also included mechanical tests, along with an antibacterial activity test to assess their practical utility as antibacterial agents. These evaluations collectively aimed to validate the efficacy of the enhancement processes and to uncover potential applications in fields such as biomedicine, pharmaceuticals, and materials science.

## 2. Experimental Section

This section elucidates the meticulous procedure employed to effectuate the surface modification of chitosan fibers through the wet impregnation method with ursolic acid and tripolyphosphate, respectively.

- 2-propanol (C_3_H_8_O) 99% Pure P.A. commercial product of Eurochem BGD from Zug, Switzerland.
- Ursolic acid (C_30_H_48_O_3_) ≥ 98% Pure P.A. commercial product of Pol-Aura from Gdańsk, Poland.
- Tripolyphosphate (TPP) (Na_5_P_3_O_10_), assay (unspecified): 90% min, Alfa Aesar from the Ward Hill, MA, USA.
- Chitosan powder commercial product of Sigma-Aldrich, from Darmstadt, Germany; molecular weight 60 kDa; degree of deacetylation (DDA) 96%.
- Acetic Acid (CH_3_COOH) 99% pure for analysis, commercial product of Poch from Gliwice, Poland.
- Sodium Hydroxide (NaOH) pure for analysis, commercial product of Poch from Gliwice, Poland.

### 2.1. Fiber Preparation

A 7% solution of chitosan was prepared in a 3.0% aqueous acetic acid solution. The solution was stirred overnight. Fibers were spun from the spinning solution on custom-made line equipment with a spinning head holding a spinneret (500 holes, 80 µm diameter each). The fibers were formed in a coagulation bath at 70 °C containing 27 g/L of sodium hydroxide in water. The fibers were one-step drawn, rinsed with water at 40 °C, and collected onto a bobbin and dried [23].

### 2.2. Fiber Impregnation Procedure for Antibacterial Properties’ Enhancement

An ursolic acid solution was prepared by dissolving the acid in pure 2-propanol at a concentration of 64 mg/mL. In order to get the chitosan fibers ready for the impregnation process, five separate samples of chitosan fibers were cut to the same size of 1.0 g each. After that, each sample was then immersed individually in 100 cm^3^ of ursolic acid solution at room temperature for varying immersion periods of 1, 2, 4, 6, and 8 h, respectively. After each immersion period, the specimens were carefully removed from the ursolic acid solution and rinsed with an aqueous solution of ethanol and distilled water 4:6 (*v*/*v*) to remove any excess ursolic acid solution, and then dried at room temperature. This process was carried out on each of the five samples.

Following the wet impregnation process, characterization and evaluation of the resulting samples were conducted to gain insight into their physical and chemical properties. The assessment encompassed a range of analytical techniques.

Fourier-Transform Infrared Spectroscopy (FTIR) was employed to investigate alterations in the chitosan fiber structure post impregnation with ursolic acid. This technique allowed for the identification of functional groups and chemical bonds, aiding in the detection of any interaction between chitosan and ursolic acid.

Scanning Electron Microscopy (SEM) was utilized to examine the morphology and surface features of the chitosan fibers. SEM provided a visual assessment of the distribution of ursolic acid on the chitosan fibers, surface topography, and potential structural modifications.

UV-Vis spectroscopy was employed to examine the fibers’ responses to ultraviolet light. This technique provided information about the fibers’ optical properties and absorption characteristics in the UV range.

To gauge the practical applicability of the modified chitosan fibers, an antibacterial activity test was carried out. This test involved exposing the modified fibers to bacteria and assessing their ability to inhibit bacterial growth. The outcomes provided critical insights into the effectiveness of the wet impregnation method in conferring antibacterial attributes to chitosan-based materials.

### 2.3. Fiber Impregnation Procedure for Mechanical Properties’ Enhancement

A solution of tripolyphosphate was prepared by dissolving it in distilled water at a concentration of 1% *w*/*v*. In order to get the chitosan fibers ready for the cross-linking process, five separate samples of chitosan fibers were cut to the same size of 1.0 g each. After that, each sample was then immersed individually in 100 cm^3^ of TPP solution at room temperature for varying immersion periods of 1, 2, 4, 6, and 8 h, respectively. After each immersion period, the specimens were carefully removed from the TPP solution and rinsed several times with distilled water to remove any excess solution. This process was repeated for each of the five specimens.

Following the immersion of chitosan fibers in the TPP solution, the resulting samples underwent comprehensive characterization and evaluation to elucidate changes in their properties. These analyses aimed to provide insights into the efficacy of the wet impregnation method, employing TPP as a cross-linker.

Fourier-Transform Infrared Spectroscopy (FTIR) was employed to investigate the chemical alterations within the chitosan fibers by comparing the spectra of the treated fibers to the spectra of untreated ones, and chemical modifications induced by the cross-linking process were discerned. Scanning Electron Microscopy (SEM) was another crucial tool utilized in this study. SEM allowed for the visualization of the fibers’ surface morphology at a microscale level. By scrutinizing the surface topography, it was possible to identify structural changes, including the formation of new interfaces or the agglomeration of TPP particles on the fiber surface. Furthermore, the mechanical properties of the chitosan fibers were assessed using dynamometry. This involved subjecting the treated fibers to mechanical testing to determine changes in their strength, elasticity, or other mechanical characteristics. Understanding these alterations was essential to ascertaining if the cross-linking process affected the fibers’ physical integrity.

### 2.4. Antibacterial Activity Test

This test was carried out by the Laboratory of Biodegradation and Microbiological Research of the Lodz Institute of Technology in accordance with ISO 20743 [34].

Briefly, 0.4 g ± 0.05 g of test and control samples underwent steam sterilization at 121 °C for 15 min. Bacterial suspensions were used to inoculate test and control samples with a density of 1.6 × 10^5^ CFU/mL of *Escherichia coli* ATCC 11229 and 2.3 × 10^5^ CFU/mL of *Staphylococcus aureus* ATCC 6538. After 24 h of incubation at 37 ± 1 °C, changes in the amount of bacteria on the test sample and on the control sample were assessed. On this basis, the antibacterial activity was calculated.

After incubation, the colonies on each plate were counted and the number of bacteria was calculated according to the formula:

$$M=\frac{\sum C}{v\times n_{1}\times d}\cdot 20$$

where:

M—the number of bacteria per sample;
C—sum of colonies in all plates from the calculated dilution;
V—volume of inoculation applied to each plate in milliliters;
n_1_—number of plates corresponding to the calculated dilution;
d—the dilution rate corresponding to the calculated dilution;
20—amount of SCDLP in milliliters used to shake out the bacteria from the sample.

The antimicrobial activity value was calculated according to the formula:

A = (lg Ct − lg C0) − (lg Tt − lg T0)

where:

lg Ct—common logarithm of the amount of bacteria on the control sample after 24 h incubation;
lg C0—common logarithm of the amount of bacteria obtained from the control sample immediately after inoculation;
lg Tt—common logarithm of the amount of bacteria obtained after 24 h incubation from the sample containing an antibacterial agent;
lg T0—common logarithm of the amount of bacteria obtained from antibacterial testing samples immediately after inoculation.

### 2.5. Tensile Strength Test

This test was performed according to ISO 2062 [35].

Following the guidelines of ISO 139 [36], the test specimens were conditioned and testing was performed at a standard atmosphere temperature of 20 °C and relative humidity of 65%. The linear density of the sample C7 Reference was 140tex and the sample after TPP treatment C7TPP was 145tex. The test length was of 250 mm for each specimen. The test was performed at a constant rate of extension of 250 mm/min. A total of 10 specimens per sample were tested.

All the tests were performed with the tensile testing machine Instron 5944 of Instron from Norwood, MA, USA and the breaking force was recorded and calculated by its own software.

## 3. Results

### 3.1. After-Fiber-Impregnation Procedure for Antibacterial Enhancement

#### 3.1.1. FTIR Spectroscopy

The infrared transmission and reflectance spectra were recorded in the range from 4000 to 600 cm^−1^ with a resolution of 4 cm^−1^ and 32 scans. Ranging from high wavenumber (4000 cm^−1^) to low wavenumber (600 cm^−1^), this allowed for the examination of both the high-energy, short-wavelength vibrations associated with functional groups like carbonyls and hydroxyls, as well as the low-energy, long-wavelength vibrations characteristic of larger molecular structures.

**Figure 1:**
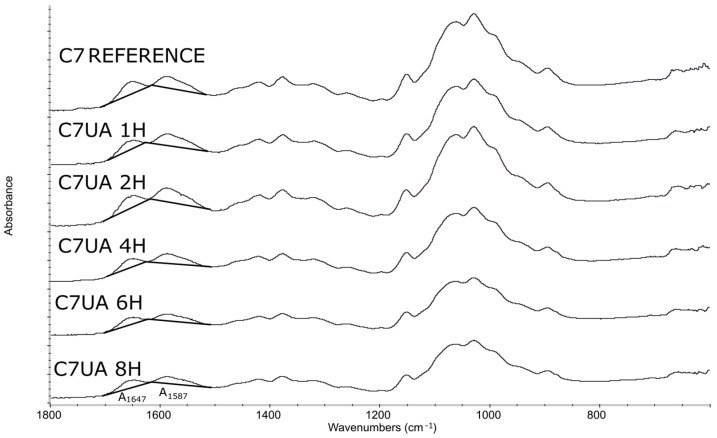
Infrared spectroscopy spectra graphs, fibers samples: reference chitosan 7% (C7 reference), chitosan 7% with ursolic acid impregnated for 1, 2, 4, 6, and 8 h (C7UA 1H, C7UA 2H, C7UA 4H, C7UA 6H, C7UA 8H).

**Figure 2:**
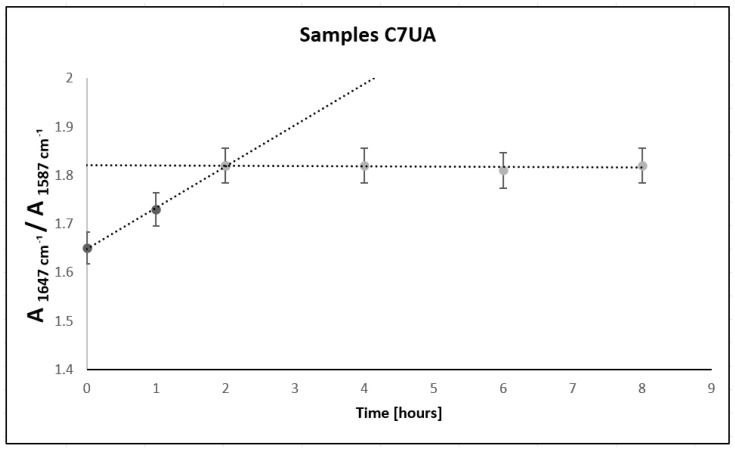
Absorbance area, amide/amine ratio for samples C7UA 0, 2, 4, 6, and 8 h.

The FTIR results of the samples C7UA immersed in ursolic acid for different time set points (0, 1, 2, 4, 6, and 8 h) (Figure 1) showed an increasing absorbance ratio from 1.66 to 1.82 over the course of 2 h. This suggested that there was ongoing physical adsorption between chitosan and ursolic acid during this time, resulting in changes to the surface of the sample. However, after 2 h, (Figure 2) the absorbance ratio remained relatively constant, suggesting that the changes had reached completion and that the chemical structure of the sample remained stable. The peaks observed at 1647 cm^−1^ and 1587 cm^−1^ are attributed to the amide and amine groups in chitosan [37], respectively. The appearance of these peaks in the FTIR spectra indicates that the chitosan fiber–ursolic acid adduct was formed through the interaction of the amine groups in chitosan with the carboxylic acid groups in ursolic acid. Following, their surface morphology was analyzed.

#### 3.1.2. Scanning Electron Microscope Images

The surface morphology of the resulting fibers was examined through microscopy, specifically employing the Scanning Electron Microscope Nova Nanosem 230 from FEI company (Hillsborough, OR, USA) for imaging and analysis. All samples were coated using the same thickness of gold, 2 nm, sputtering current (20 mA), and had the same pressure of sputtering gas (Ar), 1 Pa.

**Figure 3:**
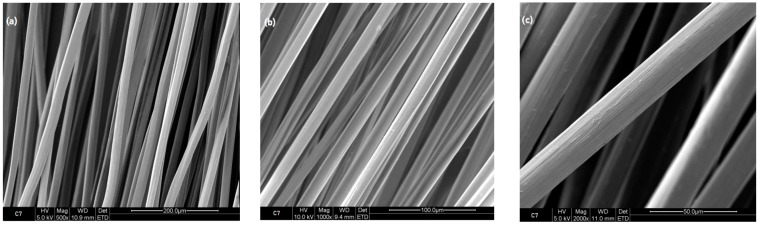
Reference fibers 7% chitosan, SEM images (**a**) 500×, (**b**) 1000×, and (**c**) 2000×.

**Figure 4:**
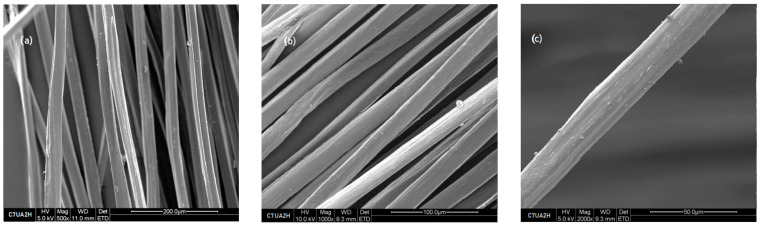
Fibers 7% chitosan with ursolic acid impregnated for 2 h, SEM images (**a**) 500×, (**b**) 1000×, and (**c**) 2000×.

When comparing the surface characteristics of the reference sample consisting of 7% chitosan (as shown in Figure 3), which remained untreated with ursolic acid, with those of the sample of 7% chitosan subjected to ursolic acid impregnation for 2 h (depicted in Figure 4), notable differences become apparent. Notably, the images captured in Figure 4 reveal an increase in the surface roughness for the latter sample. This observed variation strongly suggests that the impregnation process has effectively induced modifications of the surface topography of the chitosan fibers. Furthermore, the images derived from samples collected at different immersion periods (2, 4, 6, and 8 h) resembled the surface characteristics of the 2 h immersion sample. This uniformity in the observed surface morphology across varying immersion times suggests a consistent and stable alteration induced by the ursolic acid impregnation process.

#### 3.1.3. UV-Vis Spectroscopy

The UV-Vis spectra were obtained by means of UV-Vis from Jasco Company (Tokyo, Japan), model V-670, and the scan range used was from 190 to 400 nm with resolution 1 nm.

**Figure 5:**
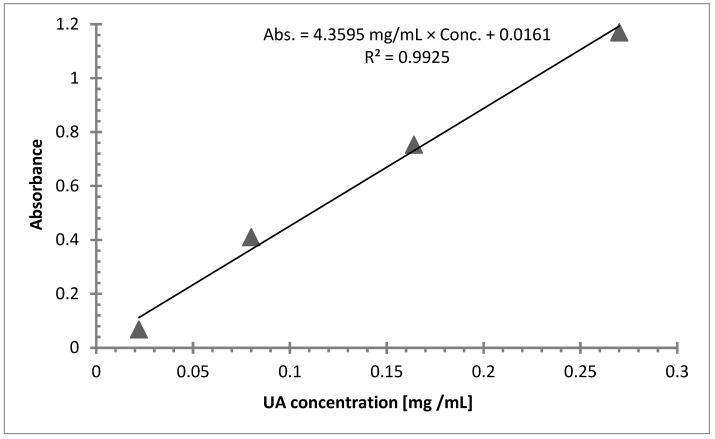
Calibration graph, relationship of absorbance/UA at concentrations of 0.0220 mg/mL, 0.080 mg/mL, 0.164 mg/mL, and 0.270 mg/mL.

Standard stock solutions containing ursolic acid were prepared in methanol at final concentrations of 2.7 mg/10 mL. After that, standard serial dilutions at three concentrations were analyzed by means of UV-Vis, and linearity was verified by regression analysis. Calibration results are presented in Figure 5 and Table 1.

**Figure 6:**
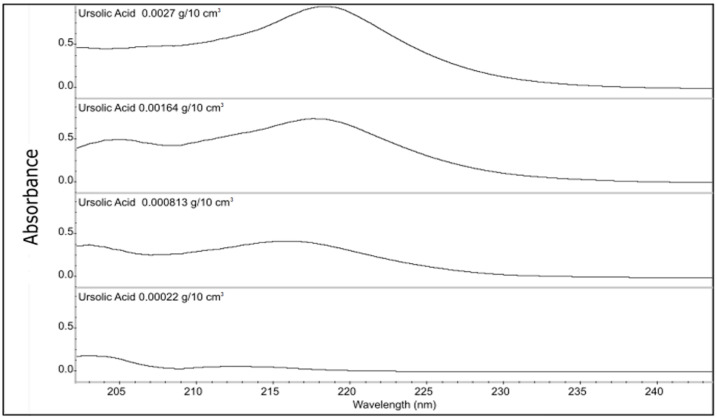
UV absorption spectra of methanolic solutions of ursolic acid analyzed at different concentrations.

The resulting spectra were analyzed at specific wavelengths to detect the distinctive peaks associated with ursolic acid. Previous research studies have reported the UV spectra of ursolic acid to display absorbance peaks between 210 nm and 220 nm [38,39]. The resulting absorbance values were obtained in ascending order and corresponded to increasing concentrations, with values of 214 nm, 216 nm, 217 nm, and 218 nm (Figure 6).

After conducting the linear analysis, a stock fiber suspension was prepared with methanol from the fibers that were impregnated with ursolic acid for 2 h at a concentration of 2.3 mg/10 mL. By adding the fibers into the methanol, it was possible to dissolve the ursolic acid deposited on the surface of the fibers in order to perform the UV-Vis test. This sample was chosen based on the FTIR test results, indicating completion of the reaction at set points in time. UV spectroscopy was performed and the absorbance peak height was appointed at 213; absorbance was 0.056, and based on the linear model, the mass of ursolic acid deposited on the fibers per gram was calculated as follows.

Abs. = 4.3595 mL/mg × Conc. + 0.0161

0.056 = 4.3595 mL/mg × Conc. + 0.0161

0.056–0.0161 = 4.3595 mL/mg × Conc.

0.0091 mg/mL = Conc. = mUAf

where mUAf = ursolic acid mass collected on the surface of 1 g of chitosan fibers.

This process was also carried out on the samples of 7% chitosan with ursolic acid that were wet-impregnated for 4, 6, and 8 h. Subsequently, the mass of ursolic acid per gram of chitosan fibers was calculated based on the knowledge that 0.0091 mg of ursolic acid is present in 2.3 mg of fibers, results are shown in Table 2.

#### 3.1.4. Antibacterial Activity Test

The antibacterial activity of the samples with 7% chitosan reference and 7% chitosan impregnated with ursolic acid for 2 h were tested and afterwards the results were later compared.

**Figure 7:**
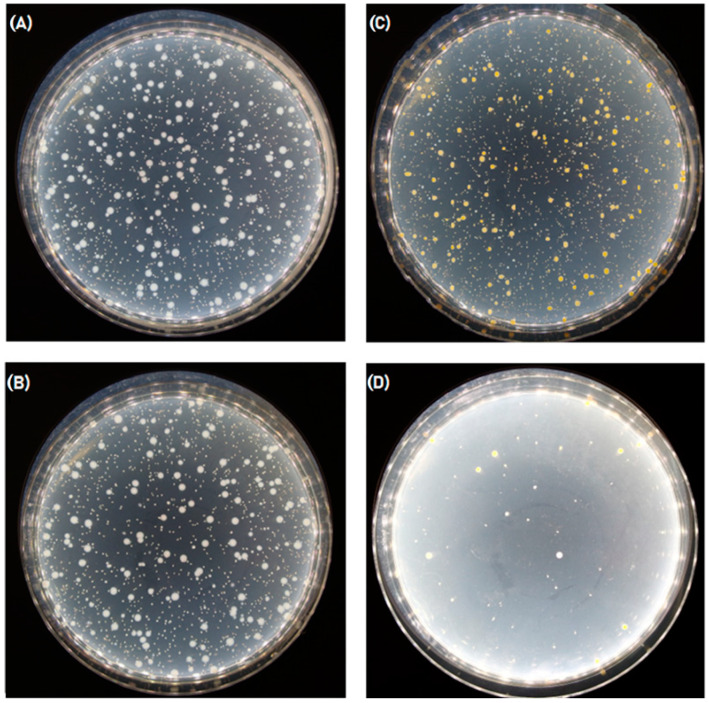
(**A**) Sample C7 reference, Petri dish with *E. Coli* culture at a dilution of 10^−1^; (**B**) sample C7UA, Petri dish with *E. coli* culture at a dilution of 10^−1^; (**C**) sample C7 reference, Petri dish with *S. aureus* culture at a dilution of 10^−1^; (**D**) sample C7UA, Petri dish with *S. aureus* culture at a dilution of 10^−1^.

After the antibacterial activity test, it was observed that the 7% chitosan reference did not exhibit any noteworthy antibacterial activity against either of the two bacterial strains (Table 3 and Table 4). The obtained value was below the efficacy threshold of A < 2 (Table 5). In contrast, the sample treated for two hours with ursolic acid showed a significant increase in antibacterial activity, with a value of 2.93 (Table 4), indicating that adding ursolic acid improved the antibacterial properties of the fibers. It is important to note that this significant antibacterial activity was only observed against the Gram-positive strain, *S. aureus*. The fact that the antibacterial activity of the fibers from the 7% chitosan sample UA 2H only displayed antibacterial activity against *S. aureus* and not *E. coli* is likely due to the difference in bacterial cell wall structure between the two organisms. Gram-positive bacteria, such as *S. aureus*, have a thick peptidoglycan layer in their cell wall that is more susceptible to damage from antibacterial agents, while Gram-negative bacteria, such as *E. coli*, have a thinner peptidoglycan layer and an additional outer membrane that provides extra protection against external agents. Therefore, it is possible that the antibacterial activity of the fibers treated with ursolic acid was not strong enough to overcome the protective mechanisms of *E. coli*, while it is effective against *S. aureus*. The overall results are presented in Table 6 and can be visually compared in Figure 7.

### 3.2. After-Fiber-Impregnation Procedure for Mechanical Enhancement

#### 3.2.1. FTIR Spectroscopy

The infrared transmission and reflectance spectra were recorded in the range from 4000 to 600 cm^−1^ with a resolution of 4 cm^−1^ and 32 scans.

**Figure 8:**
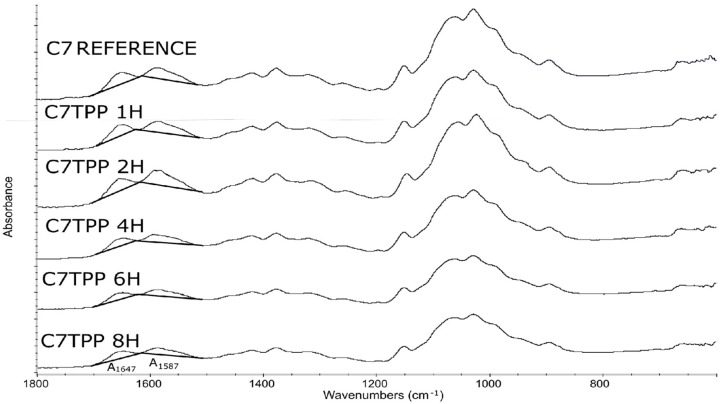
Infrared spectroscopy spectra graph, fiber samples of 7% chitosan reference, 7% chitosan. Impregnated with TPP 1, 2, 4, 6, and 8 h.

**Figure 9:**
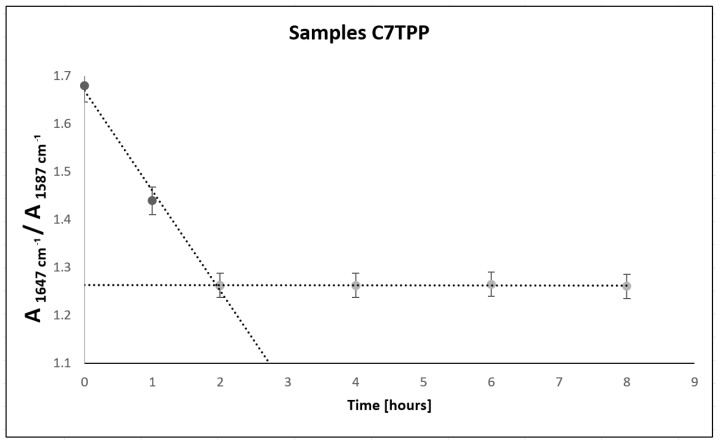
Absorbance area; amide/amine ratio for samples C7TPP 0, 1, 2, 4, 6, and 8 h.

Based on the obtained results (Figure 8), the absorbance ratio at 1647 cm^−1^/1587 cm^−1^ of samples of C7TPP at different set points of time (0, 1, 2, 4, 6, and 8 h) suggests that the cross-linking reaction between chitosan fibers and TPP ions continued up to 2 h of immersion, as indicated by the decreasing absorbance ratio. However, after 2 h of immersion, the absorbance ratio remained relatively constant (Figure 9), indicating that the reaction had reached completion. This suggests that the reaction had utilized most of the available protonated amine groups for cross-linking, and there were no more significant changes in the chemical structure of the sample during the remaining immersion time. Generally, the FTIR results suggest that protonated amine’s interaction with TPP ions was successful on the surface of chitosan fibers.

Following this, the fibers’ morphology was studied and the mechanical properties were tested to analyze if the cross-linking process improved the fibers’ tensile strength.

#### 3.2.2. Scanning Electron Microscope Images

The surface structure of the obtained fibers was analyzed microscopically using the scanning electron microscope Nova Nanosem 230.

**Figure 10:**
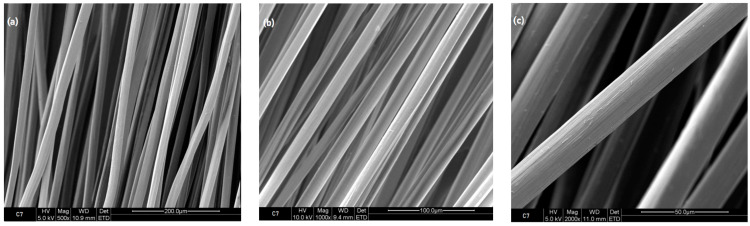
Fiber 7% chitosan reference, SEM images: (**a**) 500×, (**b**) 1000×, and (**c**) 2000×.

**Figure 11:**
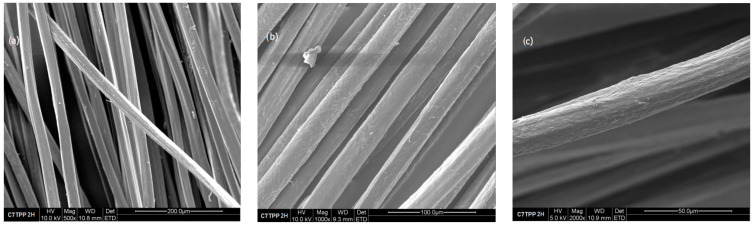
Fiber 7% chitosan impregnated with TPP for 2 h, SEM images: (**a**) 500×, (**b**) 1000×, and (**c**) 2000×.

Compared to the untreated C7 reference sample, as depicted in Figure 10, the analysis in Figure 11 reveals a distinctive change in the surface characteristics of the sample C7TPP following a 2 h immersion period. This alteration strongly indicates that the cross-linking reaction involving chitosan and TPP ions likely induced surface roughness in the fibers. Such roughness may arise from the establishment of new chemical bonds between chitosan and TPP ions, potentially leading to surface morphology deformations. Interestingly, the images captured from samples subjected to longer immersion times (4, 6, and 8 h) resembled the surface characteristics of the 2 h immersion sample. This uniformity across different immersion durations implies a consistent and stable alteration and a reaction completion resulting from the cross-linking process from 2 h of immersion, as demonstrated by the FTIR test.

#### 3.2.3. Tensile Strength Test

**Figure 12:**
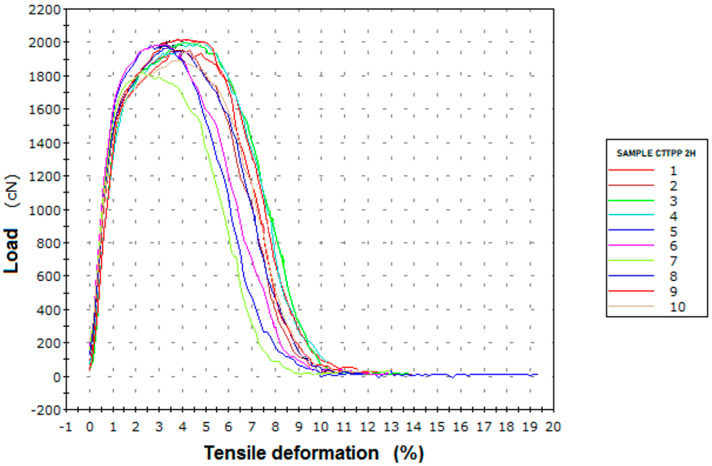
Tensile deformation graphic, sample C7TPP after 2 h of immersion.

As can be seen in Figure 12 and in Table 7, following a two-hour immersion, the sample C7TPP exhibited a noteworthy enhancement in strength when compared to the untreated C7 sample that did not undergo the wet impregnation process. This observation strongly implies the successful reinforcement of the fibers through a cross-linking reaction between chitosan and TPP. The heightened strength can be attributed to the formation of robust ionic bonds between the positively charged amino groups in chitosan and the negatively charged TPP ions, resulting in the establishment of a crosslinked network within the fibers. Furthermore, the increased relative elongation at maximum force in both samples suggests that the cross-linking reaction has concurrently imparted greater ductility to the fibers. In Table 8, the comparison of results before and after TPP impregnation at different set times is displayed, and a fiber strength improvement of up to 15.01% can be observed.

## 4. Discussion and Conclusions

The results of the present study reveal the successful surface modification of chitosan fibers 7% through wet impregnation with ursolic acid and TPP, leading to distinct enhancements in antibacterial and mechanical properties, respectively. Ursolic acid modification primarily targets antibacterial enhancement, while TPP cross-linking focuses on mechanical improvements.

The antibacterial properties of ursolic-acid-impregnated fibers demonstrate significant potential, particularly against Gram-positive strains such as *Staphylococcus aureus*. Previous works referenced in this work [23] demonstrated bacteriostatic and bactericidal action of chitosan fibers with a concentration of 5.25% and with nanosilver against both *Staphylococcus aureus* and *Escherichia coli*, but the ursolic acid–chitosan adduct has not been explored before.

The mechanical properties of the TPP cross-linked fibers were successfully enhanced, obtaining up to a 15% increase in tensile strength and up to a 9.75% increase in relative elongation. A previous work referenced in this work [19] provides valuable insights into the ionotropic cross-linking of chitosan fibers with TPP at varying pH levels. The study highlights the pH-dependent ionic cross-linking and its impact on physico-chemical properties, offering a complementary perspective to the current research, although it does not report the values of tensile strength.

The application potential of these modified fibers extends to wound dressings and scaffolds with improved antibacterial and mechanical characteristics.

In future research endeavors, the exploration of elevated concentrations of ursolic acid represents a potential route for augmenting antibacterial efficacy, with a specific focus on enhancing resistance against Gram-negative bacterial strains. This aligns with the concept of optimizing the composition to achieve a broader antimicrobial effectiveness. Furthermore, a new approach entails examining the simultaneous improvement of both antibacterial and mechanical properties in a single sample, facilitating a comprehensive understanding of the dual-functional modifications.

## Figures and Tables

**Table 1:** Linearity of calibration plots.

Linear Range (mg/mL)	y = ax + b (Linear Model)	Correlation Coefficient, R2
0.0220–0.270	Abs. = 4.3595 mg/mL × Conc. + 0.0161	0.9925

**Table 2:** Mass per gram of ursolic acid in chitosan fibers.

C7UA	Mass of UA mg/1 g of Chitosan Fibers
2 h	0.0039
4 h	0.0038
6 h	0.0040
8 h	0.0038

**Table 3:** Antibacterial activity against *Escherichia coli* ATCC 11229.

Sample ID	Incubation Time [h]	Number of Bacteria [CFU */pr]	Value of Antibacterial Activity “A”	Value of Growth
Laboratory control sample	0	3.7 × 10^4^	--------	3.37
	24	8.5 × 10^7^		
7% Chitosan reference	0	3.1 × 10^4^	0.04	3.40
	24	7.8 × 10^7^		
7% Chitosan UA 2H	0	2.8 × 10^4^	0.25	3.24
	24	4.8 × 10^7^		

Inoculum concentration: 1.6 × 10^5^ CFU/mL *E. coli*; * (CFU—colony forming unit).

**Table 4:** Antibacterial activity against *Staphylococcus aureus* ATCC 6538.

Sample ID	Incubation Time [h]	Number of Bacteria [CFU */pr]	Value of Antibacterial Activity “A”	Value of Growth
Laboratory control sample	0	9.6 × 10^4^	---------	2.13
	24	1.2 × 10^7^		
7% Chitosan reference	0	9.6 × 10^4^	0.32	2.45
	24	2.7 × 10^7^		
7% Chitosan UA 2H	0	6.7 × 10^4^	2.93	−0.68
	24	1.4 × 10^4^		

Inoculum concentration: 2.3 × 10^5^ CFU/mL *S. aureus*: * (CFU—colony forming unit).

**Table 5:** Criteria for assessing antibacterial activity.

Efficacy of Antibacterial Properties	Value of Antibacterial Activity
low	A < 2
significant	2 ≤ A < 3
strong	A ≥ 3

**Table 6:** Antibacterial activity results.

Sample ID	Value of Antibacterial Activity “A”	
	*E. coli*	*S. aureus*
C7 reference	0.32 Non-antibacterial	0.04 Non-antibacterial
C7UA	0.25 Non-antibacterial	2.93 Significant

**Table 7:** Tensile strength results, sample C7TPP after 2 h of immersion.

	Specific Strength at Maximum Force (cN/tex) Average	Relative Elongation at Maximum Force (%) Average
	14.25	3.94
Standard deviation	0.56	0.74
Coefficient of variation	2.63	10.89

Number of specimens tested: 10.

**Table 8:** Comparison of results before and after TPP impregnation.

Sample ID	Specific Strength at Maximum Force (cN/Tex) Average	Relative Elongation at Maximum Force (%) Average	Fiber’s Strength Improvement (%)	Fiber’s Elongation Improvement (%)
C7 reference	12.39	3.59	0.00	0.00
C7TPP 2 hours	14.25	3.94	15.01	9.75
C7TPP 4 hours	14.20	3.89	14.60	8.35
C7TPP 6 hours	14.22	3.87	14.76	7.80
C7TPP 8 hours	14.17	3.90	14.36	8.63

## Data Availability

Data are contained within the article.

## References

[1] Albanna M.Z., Bou-Akl T.H., Blowytsky O., Walters H.L., Matthew H.W. (2013). Chitosan fibers with improved biological and mechanical properties for tissue engineering applications. J. Mech. Behav. Biomed. Mater..

[2] Antaby E., Klinkhammer K., Sabantina L. (2021). Electrospinning of Chitosan for Antibacterial Applications—Current Trends. Appl. Sci..

[3] Fan M., Hu Q. (2017). Super Absorption Behavior of Chitosan by Freeze-Blasting in Different Alkaline Solvents. J. Renew. Mater..

[4] Geng L., Lin Y., Chen S., Shi S., Cai Y., Li L., Peng X. (2020). Superior strength and toughness of graphene/chitosan fibers reinforced by interfacial complexation. Compos. Sci. Technol..

[5] Sikorski D., Bauer M., Frączyk J., Draczyński Z. (2022). Antibacterial and Antifungal Properties of Modified Chitosan Nonwovens. Polymers.

[6] Vunain E., Mishra A.K., Mamba B.B. (2017). Fundamentals of chitosan for biomedical applications. Chitosan Based Biomaterials.

[7] Becherán L., Bocourt M., Pérez J., Peniche C., Campana S.P., Masumi M.M., Flamingo A. (2014). Chitosan in biomedicine. From gels to nanoparticles. Advances in Chitin Science, Proceeding of the 6th Iberoamerican Chitin Symposium and 12th International Conference on Chitin and Chitosan, VI SIAQ/XII ICCC, Fortaleza, Brazil, 2–5 September 2012.

[8] Ahsan S.M., Thomas M., Reddy K.K., Sooraparaju S.G., Asthana A., Bhatnagar I. (2018). Chitosan as biomaterial in drug delivery and tissue engineering. Int. J. Biol. Macromol..

[9] Rinaudo M. (2006). Chitin and chitosan: Properties and applications. Prog. Polym. Sci..

[10] Kasaai M.R. (2009). Various Methods for Determination of the Degree of N-Acetylation of Chitin and Chitosan: A Review. J. Agric. Food Chem..

[11] Li X., Feng Q., Liu X., Dong W., Cui F. (2006). Collagen-based implants reinforced by chitin fibres in a goat shank bone defect model. Biomaterials.

[12] Li X., Liu X., Dong W., Feng Q., Cui F., Uo M., Akasaka T., Watari F. (2009). In vitro evaluation of porous poly(L-lactic acid) scaffold reinforced by chitin fibers. J. Biomed. Mater. Res. Part B Appl. Biomater..

[13] Wang X., Song G., Lou T., Peng W. (2009). Fabrication of nano-fibrous PLLA scaffold reinforced with chitosan fibers. J. Biomater. Sci. Polym. Ed..

[14] Albanna M.Z., Bou-Akl T.H., Walters H.L., Matthew H.W. (2012). Improving the mechanical properties of chitosan-based heart valve scaffolds using chitosan fibers. J. Mech. Behav. Biomed. Mater..

[15] Arora B., Tandon R., Attri P., Bhatia R. (2017). Chemical Crosslinking: Role in Protein and Peptide Science. Curr. Protein Pept. Sci..

[16] Beppu M., Vieira R., Aimoli C., Santana C. (2007). Crosslinking of chitosan membranes using glutaraldehyde: Effect on ion permeability and water absorption. J. Membr. Sci..

[17] Yang Y., Chen G., Murray P., Zhang H. (2020). Porous chitosan by crosslinking with tricarboxylic acid and tuneable release. SN Appl. Sci..

[18] Muzzarelli R.A.A., El Mehtedi M., Bottegoni C., Aquili A., Gigante A. (2015). Genipin-Crosslinked Chitosan Gels and Scaffolds for Tissue Engineering and Regeneration of Cartilage and Bone. Mar. Drugs.

[19] Pati F., Adhikari B., Dhara S. (2011). Development of chitosan–tripolyphosphate fibers through pH dependent ionotropic gelation. Carbohydr. Res..

[20] Jabeen S., Saeed S., Kausar A., Muhammad B., Gul S., Farooq M. (2015). Influence of chitosan and epoxy cross-linking on physical properties of binary blends. Int. J. Polym. Anal. Charact..

[21] Tabesh E., Salimijazi H., Kharaziha M., Hejazi M. (2018). Antibacterial chitosan-copper nanocomposite coatings for biomedical applications. Mater. Today Proc..

[22] Li J., Zhuang S. (2020). Antibacterial activity of chitosan and its derivatives and their interaction mechanism with bacteria: Current state and perspectives. Eur. Polym. J..

[23] Wawro D., Stęplewski W., Dymel M., Sobczak S., Skrzetuska E., Puchalski M., Krucińska I. (2012). Antibacterial Chitosan Fibres Containing Silver Nanoparticles. Fibres Text. East. Eur..

[24] Ardean C., Davidescu C.M., Nemeş N.S., Negrea A., Ciopec M., Duteanu N., Negrea P., Duda-Seiman D., Musta V. (2021). Factors Influencing the Antibacterial Activity of Chitosan and Chitosan Modified by Functionalization. Int. J. Mol. Sci..

[25] Gadkari R.R., Ali S.W., Joshi M., Rajendran S., Das A., Alagirusamy R. (2020). Leveraging antibacterial efficacy of silver loaded chitosan nanoparticles on layer-by-layer self-assembled coated cotton fabric. Int. J. Biol. Macromol..

[26] Rinaldi F., Oliva A., Sabatino M., Imbriano A., Hanieh P.N., Garzoli S., Mastroianni C.M., De Angelis M., Miele M.C., Arnaut M. (2020). Antimicrobial Essential Oil Formulation: Chitosan Coated Nanoemulsions for Nose to Brain Delivery. Pharmaceutics.

[27] Yuan G., Lv H., Zhang Y., Sun H., Chen X. (2016). Combined Effect of Cinnamon Essential Oil and Pomegranate Peel Extract on Antioxidant, Antibacterial and Physical Properties of Chitosan Films. Food Sci. Technol. Res..

[28] Jia Z., Shen D., Xu W. (2001). Synthesis and antibacterial activities of quaternary ammonium salt of chitosan. Carbohydr. Res..

[29] Ahmadi H., Jahanshahi M., Peyravi M., Darzi G.N. (2021). A new antibacterial insight of herbal chitosan-based membranes using thyme and garlic medicinal plant extracts. J. Clean. Prod..

[30] Ghasemzadeh F., Najafpour G.D., Mohammadi M. (2021). Antiinfective properties of ursolic acid-loaded chitosan nanoparticles against *Staphylococcus aureus*. Turk. J. Chem..

[31] Wolska K., Grudniak A., Fiecek B., Kraczkiewicz-Dowjat A., Kurek A. (2010). Antibacterial activity of oleanolic and ursolic acids and their derivatives. Cent. Eur. J. Biol..

[32] Do Nascimento P.G.G., Lemos T.L.G., Bizerra A.M.C., Arriaga M.C., Ferreira D.A., Santiago G.M.P., Braz-Filho R., Costa J.G.M. (2014). Antibacterial and Antioxidant Activities of Ursolic Acid and Derivatives. Molecules.

[33] Cunha W.R., de Matos G.X., Souza M.G.M., Tozatti M.G., Andrade e Silva M.L., Martins C.H., da Silva R., Da Silva Filho A.A. (2010). Evaluation of the antibacterial activity of the methylene chloride extract of *Miconia ligustroides*, isolated triterpene acids, and ursolic acid derivatives. Pharm. Biol..

[34] (2021). Textiles—Determination of Antibacterial Activity of Textile Products.

[35] (2009). Textiles—Yarns from Packages—Determination of Single-End Breaking Force and Elongation at Break Using Constant Rate of Extension (CRE) Tester.

[36] (2005). Textiles—Standard Atmospheres for Conditioning and Testing.

[37] Lin H.-Y., Yeh C.-T. (2010). Controlled release of pentoxifylline from porous chitosan-pectin scaffolds. Drug Deliv..

[38] Olszewska M. (2008). Optimization and validation of an HPLC-UV method for analysis of corosolic, oleanolic, and ursolic acids in plant material: Application to *Prunus serotina* Ehrh. Acta Chromatogr..

[39] Wei M.-C., Yang Y.-C. (2014). Extraction characteristics and kinetic studies of oleanolic and ursolic acids from *Hedyotis diffusa* under ultrasound-assisted extraction conditions. Sep. Purif. Technol..

